# Headache: an important factor associated with muscle soreness/pain at the two-year follow-up point among patients with major depressive disorder

**DOI:** 10.1186/s10194-016-0648-3

**Published:** 2016-05-27

**Authors:** Ching-I Hung, Chia-Yih Liu, Ching-Hui Yang, Shuu-Jiun Wang

**Affiliations:** Department of Psychiatry, Chang Gung Memorial Hospital, Linkou, Taiwan; Chang Gung University College of Medicine, Tao-Yuan, Taiwan; Department of Nursing, Chang Gung University of Science and Technology, Tao-Yuan, Taiwan; Faculty of Medicine, National Yang-Ming University School of Medicine and Neurological Institute, Taipei Veterans General Hospital, Taipei, Taiwan; Department of Neurology, Taipei Veterans General Hospital, No. 201 Shi-Pai Road, Section 2, Taipei, 112 Taiwan

**Keywords:** Depression, Anxiety, Migraine, Pain, Muscle soreness

## Abstract

**Background:**

No study has compared the associations of headache, anxiety, and depression at baseline with muscle soreness or pain (MS/P) at baseline and at the two-year follow-up point among outpatients with major depressive disorder (MDD). This study aimed to investigate the above issue.

**Methods:**

This study enrolled 155 outpatients with MDD at baseline, and 131 attended a two-year follow-up appointment. At baseline, migraine was diagnosed based on the *International Classification of Headache Disorders*, 2^nd^ edition. MDD and anxiety disorders were diagnosed using the Structured Clinical Interview for DSM-IV-TR. The visual analog scale was used to evaluate the intensities of headache and MS/P in the neck, shoulder, back, upper limbs, and lower limbs. Depression and anxiety were evaluated using the Hospital Anxiety and Depression Scale. Multiple linear regressions were used to compare the associations of these factors with MS/P.

**Results:**

Compared with anxiety disorders, migraine was more strongly associated with MS/P in all areas at baseline and in the upper and lower limbs at follow-up. Headache intensity at baseline was the factor most strongly associated with MS/P in all areas at baseline and follow-up after controlling for depression and anxiety. Headache intensity at baseline predicted MS/P at baseline and follow-up.

**Conclusions:**

Migraine and headache intensity are important factors related to MS/P at baseline and follow-up among patients with MDD. Integrating depression and headache treatment might be indicated to improve MS/P.

## Background

Depression and muscle soreness or pain (MS/P) are closely related and interact [1–3]. Chronic muscle pain is frequently accompanied by symptoms of depression [2]. Major depressive disorder (MDD) is common among chronic pain patients with myofascial pain syndrome [4]. Co-occurrence of musculoskeletal pain and depressive symptoms is strongly related to poor self-rated physical work ability [3]. Among patients with depression, muscle soreness is one of the factors that independently predict a slower remission after controlling for the baseline severity of depression [5].

Headache, depression, and anxiety are closely related and interact [6–8]. A strong bidirectional association between migraine and psychiatric disorders has been documented [8]. Migraine is common among patients with MDD and bipolar disorders [9–11]. Migraine is related to poorer recovery of health-related quality of life after acute pharmacotherapy among patients with MDD [12]. MDD patients with migraine are associated with greater severities of depression, anxiety, and somatic and pain symptoms [9, 10, 13, 14]. Migraine is also associated with an increased frequency of suicidal ideation and suicide attempts in patients with MDD [8]. Migraine and other headaches in a general population are associated with increased musculoskeletal symptoms [15].

Anxiety is associated with musculoskeletal symptoms [1, 16]. Increased muscle tension is one criterion for generalized anxiety disorder (GAD) [17]. Fifty percent of workers with persistent musculoskeletal pain undergoing work rehabilitation initially exhibited GAD symptoms [18]. Anxiety and/or depression are factors related to poor outcomes and disability related to musculoskeletal pain [19, 20]. Anxiety, depression, and migraine were associated with pain in the back, neck, orofacial area, abdomen, joints, and chest, but the associations of migraine with the six pains weakened substantially after correction for the severity of anxiety and depression [21]. Therefore, a considerable part of the comorbidity of migraine and the six pains might be explained by depression and anxiety [21].

As mentioned above, MS/P, migraine, depression, and anxiety are closely related and interact. Investigation of factors related to MS/P among patients with MDD is important, because MS/P is related to a poor treatment prognosis of depression and associated with occupational disability [3, 5, 20]. Although previous studies have reported associations of migraine or other headaches with MS/P among patients with headaches or in a general population [15, 22, 23], the associations of migraine or headaches with MS/P among patients with MDD have been neglected. No study has compared the associations of migraine and anxiety disorders with MS/P in addition to comparing the associations of headache indices, depression, and anxiety with MS/P at baseline and during long-term follow-up among patients with MDD.

The aims of this study were to compare the associations of headaches, anxiety, and depression evaluated at baseline with MS/P at baseline and at the two-year follow-up point among patients with MDD.

We hypothesized that the association of headaches with MS/P is not inferior to the associations of anxiety and depression with MS/P after controlling for demographic and other variables.

## Methods

### Subjects

The study was approved by the Institutional Review Board of Chang Gung Memorial Hospital, a medical center in northern Taiwan. This project was conducted in the psychiatric outpatient clinics of the same hospital from September 2005 to August 2009. Written informed consent, based on the guidelines regulated in the Declaration of Helsinki, was obtained from all subjects prior to their entrance into the study.

Study participants, aged 18–65 years, were recruited from consecutive outpatients who met the DSM-IV- text revision (TR) criteria for MDD [17] and were experiencing a current major depressive episode (MDE) and had not taken antidepressants or other psychotropic drugs within the previous four weeks. Three exclusion criteria were established in order to prevent depression and pain symptoms from being confounded by psychotic symptoms, substance abuse, or other medical conditions: 1) psychotic symptoms, severe psychomotor retardation, mental retardation, or catatonic features; 2) a history of substance dependence or abuse without full remission in the previous month; and 3) chronic medical diseases such as diabetes mellitus, hypertension, severe neurological disorders, and other medical diseases, except for headaches. The Structured Clinical Interview for DSM-IV-TR Axis I Disorders was used to diagnose MDD and anxiety disorders [24].

### Assessment of headaches

A structured headache intake form was designed to meet the operational criteria of the *International Classification of Headache Disorders*, 2nd edition (ICHD-2) [25]. Questions regarding headache pattern, intensity, frequency, location, duration, aggravation by physical activities, aura, nausea, vomiting, phonophobia, photophobia, precipitating factors and medication usage for headache were included. At baseline, subjects completed the structured headache intake form. An experienced headache specialist, who was masked to the results of psychiatric evaluations, interviewed all patients after they had completed the headache intake form and made diagnoses. Patients who fulfilled the criteria of migraine without aura and/or migraine with aura were categorized as the “migraine” group, while the other subjects were categorized as the “non-migraine” group.

The average headache intensity in the past week was evaluated using a visual analog scale (VAS), with 0 representing “no pain” and 10 representing “pain as severe as I can imagine”. The number of headache days in the past week was recorded as the headache frequency. The intensity and frequency were evaluated at baseline and at two-year follow-up.

### Assessment of anxiety disorders

One board-certified psychiatrist, who was masked to the diagnoses of headaches and the psychometric results, used the Structured Clinical Interview to diagnose the following anxiety disorders: panic disorder, agoraphobia, specific phobia, social phobia, obsessive–compulsive disorder, post-traumatic stress disorder, and GAD [24]. Patients with any one of the anxiety disorders in a current episode or partial remission were categorized as the “anxiety disorders” group, while the others were classified as the “non-anxiety disorders” group.

### Assessment of muscle soreness or pain, depression, and anxiety

The average MS/P in the past week was evaluated by using a VAS, with 0 representing “no pain” and 10 representing “pain as severe as I can imagine”. MS/P in five areas was evaluated, including the neck, shoulder, back, upper limbs, and lower limbs.

The severities of depression and anxiety were evaluated by the Hospital Anxiety and Depression Scale (HADS). The HADS includes 7 items for the anxiety subscale (HADS-A) and 7 for the depression subscale (HADS-D), with the scores for each subscale ranging from 0 to 21 [26, 27]. The HADS was used because it does not include any somatic or pain symptoms, which might be related to MS/P. The HADS and MS/P were measured at baseline and follow-up.

### Procedures

After enrollment, subjects were treated for four weeks with venlafaxine extended-release, one 75 mg capsule per day, and zolpidem. After the four-week treatment, these patients were treated as general psychiatric outpatients without controlling their pharmacotherapy. In the following two years, some patients continued pharmacotherapy and others dropped out. Two years later, the subjects were followed-up. Subjects with pharmacotherapy in the index follow-up month were categorized into the treated group and those without were categorized into the not treated group.

### Statistical methods

All statistical analyses were performed using SPSS for Windows 12.0 (SPSS Inc., Chicago, IL, USA). The independent *t* test, the Mann-Whitney *U* test, the paired *t* test, Pearson’s correlation, Spearman’s correlation and the Chi-square test were used as appropriate.

Two models of multiple linear regressions with forward selection were used to compare the associations of these independent variables with MS/P in the five areas at baseline and two-year follow-up after controlling for other factors. The first regression model compared the associations of two categorical variables (migraine and anxiety disorders) diagnosed at baseline with MS/P in the five areas at baseline and at the two-year follow-up point. The second model compared the associations of four continuous variables (headache intensity, headache frequency, HADS-D score and HADS-A score) evaluated at baseline with MS/P in the five areas at baseline and at the two-year follow-up point. In the first and second models, the dependent variables were MS/P (VAS scores) in the five areas at baseline and follow-up. In the first model, the independent variables included five demographic variables (gender, age, marital status, educational years, and employment status), with pharmacotherapy or not at follow-up, with migraine or not at baseline, and with anxiety disorders or not at baseline. In the second model, the independent variables included five demographic variables, the four continuous variables (headache intensity, headache frequency, HADS-D score and HADS-A score) at baseline, and whether or not pharmacotherapy is being used at follow-up.

A two-tailed *P* value <0.05 was considered statistically significant. In the comparison of MS/P in the five areas between groups, Bonferroni correction was used and a two-tailed *P* value <0.01 was considered to indicate a significant difference.

## Results

### Subjects

At baseline, 155 patients were enrolled (Table 1). At the two-year follow-up, 131 patients agreed to participate in the follow-up, 11 patients refused to participate, and 13 patients were unable to be contacted by phone and mail. There were no significant differences in the five demographic variables, the percentages of migraine and anxiety comorbidities, the HADS scores, or the headache indices between the patients who were followed-up and those who were not followed-up.

**Table 1:** Demographic variables and comorbidities among patients with major depressive disorder

	Baseline	Two years
Case number	155	131
Age (years)	30.3 ± 8.0	32.5 ± 8.3
Educational years	13.4 ± 2.5	13.5 ± 2.5
Female (%)	68.4	66.4
In employment (%)	59.4	58.8
Married (%)	38.7	40.5
With pharmacotherapy (%)	–	22.9
With migraine (%)	47.1	46.6
With anxiety disorders (%)	45.2	45.8
HADS-D scores^a^	14.4 ± 3.4	7.1 ± 5.3
HADS-A scores^a^	14.8 ± 3.4	8.7 ± 4.6
Headache intensity^b^	4.2 ± 3.1	2.1 ± 2.6
Headache frequency^c^	3.4 ± 2.6	2.2 ± 2.5

HADS-D the depression subscale of the Hospital Anxiety and Depression Scale (HADS), HADS-A the anxiety subscale of the HADS

^a^50.3 and 56.1 % of the subjects had severe depression and anxiety at baseline, respectively, with HADS-D and HADS-A scores ranging from 15 to 21

^b^The average headache intensity, measured using a visual analog scale, in the past week

^c^The headache days in the past week

At the two-year follow-up, of 30 patients in the treated group, 18 (60.0 %) and 12 were treated with venlafaxine (mean dosage of 102.1 ± 49.5 mg per day) and other antidepressants, respectively. Benzodiazepine or other hypnotics were used in 19 (63.3 %) subjects.

### Headache and psychiatric diagnoses

Among the 155 subjects at baseline, 73 (47.1 %) had migraine, including 16 subjects with chronic migraine, 2 with chronic migraine and medication overuse headache, 49 with episodic migraine without aura, and 6 with episodic migraine both with and without aura. The other 82 participants included 27 with probable migraine without aura, 1 with chronic tension-type headache (TTH), 30 with episodic TTH, 6 with probable episodic TTH, 9 with headache unspecified, and 9 without any headaches.

At baseline, 70 (45.2 %) patients had at least one anxiety disorder, including 15 (9.7 %) with panic disorder and/or agoraphobia, 38 (24.5 %) with social phobia, 28 (18.1 %) with specific phobia, 18 (11.6 %) with post-traumatic stress disorder, 10 (6.5 %) with obsessive-compulsive disorder, and 11 (7.1 %) with GAD.

MDD subjects with migraine had a higher risk of comorbidity with any one of the anxiety disorders (63.4 % vs. 28.0 %, odds ratio = 4.64, *P* <0.001) as compared with MDD subjects without migraine.

### Differences in the severities of MS/P between groups

Table 2 shows the severities of MS/P at baseline and follow-up. For the full sample at baseline and follow-up, there was a trend that MS/P in the shoulder was of the greatest severity, followed by the neck, back, and limbs. At baseline, the patients with migraine had significantly (post-Bonferroni correction; *P* <0.01) greater severities of MS/P in the five areas as compared with the patients without migraine. Compared with patients without anxiety disorders, patients with anxiety disorders also had significantly (*P* <0.01) higher pain intensities in the five areas, except for neck soreness or pain.

**Table 2:** The intensities of muscle soreness/pain between groups among patients with major depressive disorder at baseline and follow-up^a^

Time points	Treatment	Comorbidities at baseline^b^		Number	Neck	Shoulder	Back	Upper limbs	Lower limbs
Baseline		Full sample		155	5.0 ± 3.5	5.1 ± 3.6	3.8 ± 3.6	2.6 ± 3.2	2.3 ± 3.0
	No Tx	Migraine	Yes	73	6.4 ± 3.2**	6.8 ± 3.2**	5.1 ± 3.7**	3.5 ± 3.4**	3.3 ± 3.2**
	No Tx	Migraine	No	82	3.7 ± 3.3	3.7 ± 3.3	2.6 ± 3.1	1.7 ± 2.8	1.4 ± 2.4
	No Tx	Anxiety	Yes	70	5.7 ± 3.5*	6.1 ± 3.5**	4.6 ± 3.9**	3.4 ± 3.4**	3.1 ± 3.3**
	No Tx	Anxiety	No	85	4.4 ± 3.5	4.3 ± 3.5	3.1 ± 3.2	1.9 ± 2.9	1.7 ± 2.6
Two years	–	Full sample		131	3.0 ± 3.2	3.3 ± 3.1	2.0 ± 2.8	1.3 ± 2.3	1.5 ± 2.3
	No Tx	Migraine	Yes	43	3.2 ± 3.6	3.9 ± 3.5	2.6 ± 3.3	1.9 ± 3.0*	2.0 ± 2.8*
	No Tx	Migraine	No	58	2.8 ± 3.0	2.8 ± 2.8	1.8 ± 2.6	0.8 ± 1.4	1.1 ± 1.6
	No Tx	Anxiety	Yes	45	3.4 ± 3.5	4.0 ± 3.4*	2.7 ± 3.3	1.9 ± 3.0*	2.0 ± 2.8*
	No Tx	Anxiety	No	56	2.7 ± 3.0	2.7 ± 2.8	1.7 ± 2.5	0.7 ± 1.4	1.0 ± 1.6
	Tx	Migraine	Yes	17	3.7 ± 2.8*	4.3 ± 2.9	2.2 ± 2.4	1.7 ± 2.6	2.1 ± 2.9
	Tx	Migraine	No	13	1.5 ± 3.0	2.0 ± 3.1	1.2 ± 2.5	1.1 ± 1.8	1.2 ± 2.3
	Tx	Anxiety	Yes	16	3.7 ± 3.2	4.0 ± 3.3	1.9 ± 2.7	1.3 ± 2.0	1.9 ± 2.7
	Tx	Anxiety	No	14	1.7 ± 2.6	2.5 ± 3.0	1.7 ± 2.2	1.5 ± 2.7	1.5 ± 2.6

Tx accepted pharmacotherapy in the index follow-up month

^a^The independent *t* test and the Mann-Whitney *U* test were used where appropriate

^b^Anxiety comorbidities represented patients with at least one of following anxiety disorders: panic disorder, agoraphobia, social phobia, specific phobia, post-traumatic stress disorder, obsessive–compulsive disorder, and generalized anxiety disorder

*P < 0.05; **P < 0.01

At the two-year follow-up point, differences in MS/P in all areas post-correction were not significant between patients with and without migraine or between patients with and without anxiety disorders, both in the treated and not treated groups.

At the two-year follow-up point, the severities of MS/P in the five areas, HADS-A, HADS-D, and headache indices had significantly (*P* <0.05) decreased both in the treated and not treated groups compared with these variables at baseline, except for the severities of MS/P in the upper and lower limbs and two headache indices in the treated group.

### The correlations of MS/P with headache indices, depression, and anxiety

Table 3 shows the correlations of MS/P at baseline and follow-up with headache indices, depression, and anxiety at baseline. At baseline, the correlations of MS/P in all areas with headache intensity, headache frequency, and HADS-A scores were significant. However, the correlations of MS/P with the HADS-D scores were not significant, except for MS/P in the shoulder.

**Table 3:** The correlations of muscle soreness/pain at baseline and two-year follow-up with headache indices, depression, and anxiety at baseline

Time points	Treatment	Number		Neck	Shoulder	Back	Upper limbs	Lower limbs
Baseline	No Tx	155	HI	0.46**	0.45**	0.36**	0.29**	0.37**
	No Tx	155	HF	0.36**	0.36**	0.20*	0.16*	0.16*
	No Tx	155	HADS-D	0.12	0.17*	0.12	0.01	0.08
	No Tx	155	HASD-A	0.26**	0.32**	0.33**	0.17*	0.19*
Two years	No Tx	101	HI	0.13	0.31**	0.21*	0.26**	0.27**
	No Tx	101	HF	0.02	0.21*	0.12	0.15	0.14
	No Tx	101	HADS-D	−0.02	−0.03	−0.04	−0.02	−0.05
	No Tx	101	HASD-A	0.01	0.07	0.06	0.11	0.21*
	Tx	30	HI	0.56**	0.41*	0.45*	0.32	0.39*
	Tx	30	HF	0.42*	0.33	0.31	0.34	0.44*
	Tx	30	HADS-D	0.08	0.03	−0.15	0.03	0.20
	Tx	30	HASD-A	0.22	0.08	−0.02	−0.24	−0.07

HADS-D the depression subscale of the Hospital Anxiety and Depression Scale (HADS), HADS-A the anxiety subscale of the HADS, HI average headache intensity in the past week, HF headache frequency measured by the headache days in the past week, Tx accepted pharmacotherapy in the index follow-up month

*P < 0.05; **P < 0.01

At the two-year follow-up point, the headache intensity at baseline was significantly correlated with MS/P in all areas in the treated and not-treated group, except for MS/P in the neck in the not-treated group and in the upper limbs in the treated group. Headache frequency at baseline was significantly correlated with MS/P in the shoulder in the not-treated group and MS/P in the neck and lower limbs in the treated group. The correlations of HADS-D and HADS-A scores with MS/P in all areas were not significant in both groups, except for the correlation of HADS-A with MS/P in the lower limbs in the not-treated group.

### Comorbidities independently associated with MS/P

In the first regression model (Table 4), migraine at baseline was an independent factor related to MS/P in the all areas at baseline after controlling for demographic variables. At the two-year follow-up point, migraine at baseline was also an independent factor related to MS/P in the upper and lower limbs. A trend was noted that the association of migraine with MS/P had decreased at the two-year follow-up point. Anxiety disorder at baseline was an independent factor related to MS/P in the shoulder at the two-year follow-up.

**Table 4:** Independent comorbidities associated with muscle soreness/pain among patients with major depressive disorder^a,b^

Areas	Independent variable	Beta	*R* ^*2*^ change	*t*	*P*
Neck_(B)_	Migraine_(B)_	0.40	0.16	5.3	<0.01
Neck_(2Y)_	No	–	–	–	–
Shoulder_(B)_	Migraine_(B)_	0.43	0.19	6.0	<0.01
Shoulder_(2Y)_	Anxiety disorders_(B)_	0.21	0.05	2.5	0.01
Back_(B)_	Migraine_(B)_	0.35	0.12	4.6	<0.01
Back_(2Y)_	No	–	–	–	–
Upper limbs_(B)_	Migraine_(B)_	0.27	0.07	3.5	<0.01
Upper limbs_(2Y)_	Migraine_(B)_	0.22	0.05	2.6	0.01
Lower limbs_(B)_	Migraine_(B)_	0.32	0.10	4.2	<0.01
Lower limbs_(2Y)_	Migraine_(B)_	0.21	0.04	2.4	0.02

^a^The case numbers were 155 and 131 at baseline and two-year follow-up, respectively

^b^“(B)” and “(2Y)” were used to represent the variable evaluated at baseline and two-year follow-up, respectively

### Headache, depression, and anxiety independently associated with MS/P

In the second regression model (Table 5), headache intensity was the most important factor (with the greatest R square change) related to MS/P in all areas at baseline and follow-up after controlling for other factors. Headache frequency did not enter these regressions.

**Table 5:** Independent factors associated with muscle soreness/pain among patients with major depressive disorder^a,b^

Areas	Independent variable	Beta	*R* ^*2*^ change	*t*	*P*
Neck_(B)_	HI_(B)_	0.42	0.21	5.8	<0.01
	HADS-A_(B)_	0.16	0.02	2.2	0.03
Neck_(2Y)_	HI_(B)_	0.22	0.05	2.6	0.01
Shoulder_(B)_	HI_(B)_	0.38	0.20	5.3	<0.01
	HADS-A_(B)_	0.24	0.05	3.3	<0.01
	gender_(B)_	−0.17	0.03	−2.4	0.02
Shoulder_(2Y)_	HI_(B)_	0.33	0.11	4.0	<0.01
Back_(B)_	HI_(B)_	0.29	0.13	3.9	<0.01
	HADS-A_(B)_	0.27	0.06	3.6	<0.01
	gender_(B)_	−0.15	0.02	−2.1	0.04
Back_(2Y)_	HI_(B)_	0.25	0.06	3.0	<0.01
Upper limbs_(B)_	HI_(B)_	0.29	0.09	3.8	<0.01
Upper limbs_(2Y)_	HI_(B)_	0.27	0.08	3.2	<0.01
Lower limbs_(B)_	HI_(B)_	0.37	0.14	4.9	<0.01
Lower limbs_(2Y)_	HI_(B)_	0.30	0.09	3.5	<0.01

HADS-A the anxiety subscale of the Hospital Anxiety and Depression Scale, HI = average headache intensity in the past week

^a^The case numbers were 155 and 131 at baseline and two-year follow-up, respectively

^b^“(B)” and “(2Y)” were used to represent the variable evaluated at baseline and two-year follow-up, respectively

Anxiety (HADS-A score) was a factor independently associated with MS/P in three areas at baseline. However, anxiety was not a significant factor related to MS/P in all areas at follow-up. Depression (HADS-D score) was not a significant factor related to MS/P in all areas at baseline and follow-up.

## Discussion

Migraine at baseline was an independent factor related to MS/P after controlling for other factors. The association of migraine with MS/P was not limited to baseline measurements, but persisted to the two-year follow-up point in the upper and lower limbs. Previous studies have reported that migraine is associated with increased musculoskeletal symptoms and increased self-reported muscle tension [15, 23, 28]. This might result from the fact that migraine is related to increased intracranial and extracranial mechanical sensitivities and associated with somatosensory amplification [29, 30]. Repeated migraine attacks might cause central sensitization, which is related to allodynia, hyperalgesia and spontaneous pain [29, 31]. Therefore, MDD patients with migraine might become more sensitive to other pains. One previous study reported that the impact of migraine on physical and pain symptoms might be greater than that of a MDE among psychiatric outpatients [10]. The upper and lower limbs were the areas continuously associated with migraine at the two-year follow-up point. Previous studies have reported that limb pain is associated with migraine and has been considered part of migrainous syndrome [32–34]. In this study, migraine was more associated with MS/P in most areas than anxiety disorders. Therefore, clinicians should not ignore migraine when treating MS/P in patients with MDD.

Headache intensity at baseline was the factor most strongly associated with MS/P at baseline and at the two-year follow-up point. This demonstrated that the association of headache intensity with MS/P was greater than the associations of the severities of depression and anxiety with MS/P. Previous studies have reported that headache is related to musculoskeletal symptoms [15, 23]. Therefore, headache might be an important factor related to central sensitization among patients with MDD. Our results implied that headache intensity at baseline might be an important marker related to MS/P among patients with MDD. MDD patients with a greater intensity of headache during a MDE might have greater severities of MS/P at long-term follow-up. However, further evidence may be needed in order to prove these hypotheses.

Three points were worthy of note. 1) One longitudinal population-based cohort study found a bidirectional relationship between headache and chronic musculoskeletal complaints [22]. Therefore, the pathophysiology of headache and chronic musculoskeletal complaints might overlap or be closely related. The study concluded that chronic musculoskeletal complaints should be treated not only to relieve them but also to prevent the development of chronic daily headache, and vice versa [22]. This conclusion hinted that treatment of migraine and headache might be helpful to improve MS/P among patients with MDD. 2) Depression, migraine, and fibromyalgia are often comorbid with each other and interact [9, 10, 35–37]. Our study showed that migraine and headache intensity were factors related to MS/P, an important characteristic of fibromyalgia [38], among patients with MDD. Our study provided further evidence of correlations between depression, migraine, headache, and fibromyalgia. 3) Our study demonstrated that headache intensity was associated with MS/P to a greater extent than headache frequency. One previous study reported that frequency of headache had a higher impact than headache diagnosis on the association between headache and musculoskeletal symptoms [15]. The impact ranking of headache intensity, headache frequency, and headache diagnoses associated with musculoskeletal symptoms should be further investigated.

Several limitations or methodological issues should be addressed. 1) In the clinical neutral study, subjects in the treated and not treated groups were not divided by randomization, but by patients’ decisions. This might cause bias. Moreover, the division caused small sample sizes, which hindered assessment of the statistical significance. 2) Although pharmacotherapy was listed as an independent factor in the regression model, the content of pharmacotherapy was not controlled at follow-up. Analysis of the impacts of different medications on MS/P would be hindered by the small sample sizes. 3) The headache intensity and frequency in the past week were recalled by subjects. A more reliable method would be to record headache parameters in a prospective headache diary. 4) Our sample was collected from a medical center. Expansion of the results of this study to the general population should be performed cautiously. 5) This study only focused on the association of migraine with MS/P. Some patients with migraine might have comorbidity with TTH, which is also related to muscle tension. The association of TTH with MS/P should be investigated in future studies.

## Conclusion

Compared with anxiety disorders, migraine was more strongly associated with MS/P in all areas at baseline and in the upper and lower limbs at the two-year follow-up point among patients with MDD. Headache intensity was the factor most strongly associated with MS/P in all areas at baseline and follow-up after controlling for depression, anxiety, and other factors. Headache intensity evaluated at baseline had impacts on MS/P in all areas not only at baseline, but also at the two-year follow-up point. Migraine and headache intensity should not be neglected in the treatment of MS/P in patients with MDD. Simultaneously, treatment of depression and headache might improve MS/P.

## Abbreviations

GAD, generalized anxiety disorder; HADS, Hospital Anxiety and Depression Scale; HADS-A, anxiety subscale of the HADS; HADS-D, depression subscale of the HADS; ICHD-2, International Classification of Headache Disorders 2nd edition; MDD, major depressive disorder; MDE, major depressive episode; MS/P, muscle soreness or pain; TTH, tension-type headache, VAS visual analog scale.
